# Prevalence of Malnutrition among Elderly People Living in a Rural Area of Nepal

**DOI:** 10.31729/jnma.6013

**Published:** 2021-02-28

**Authors:** Jagdish Chataut, Shristi Jonche, Manish Ghimire, Dipesh Tamrakar, Mukta Singh Bhandari

**Affiliations:** 1 Department of Community Medicine, Kathmandu University School of Medical Sciences, Dhulikhel, Kavre, Nepal

**Keywords:** *body mass index*, *elderly*, *nutritional status.*

## Abstract

**Introduction:**

Elderly population is more likely to suffer from malnutrition due to aging-associated factors that influence nutritional status like loss of appetite, swallowing difficulties, digestive problems, and chronic illness. There is insufficient information related to the nutritional status of the elderly in Nepal. Hence, this study aims to determine the prevalence of malnutrition among elderly people living in the rural area of the Kavrepalanchok district.

**Methods:**

A descriptive cross-sectional study was conducted among 320 elderly people aged ≥60 years in a rural area of Kavre district from August to October 2019. Ethical approval was taken from the Institutional Review Committee (IRC-KUSMS: 68/19). Convenient sampling was done. Data analysis was performed using the Statistical Program for Social Sciences version 23.

**Results:**

The prevalence of malnutrition and risk of malnutrition was 37 (11.6%) and 159 (49.7%), respectively. Of 320 elderly persons, 193 (60.3%) males and 127 (39.7%) females, with a mean age of 68.23±7.38 years, participated in this study. The mean BMI was 22.54±3.25 kg/m^2^ (Mean ± SD).The prevalence of malnutrition was higher among females 19 (15%) compared to males 18 (9.3%).

**Conclusions:**

The prevalence of malnutrition and risk of malnutrition is high in the study population. Interventions to improve the nutritional status of the elderly should focus primarily on older people, females, and those who have co-morbidities.

## INTRODUCTION

Globally the proportion of elderly people is constantly increasing and 79% of them will be residing in developing regions.^1,2^ The Nepali Senior Citizens Act defines the elderly population as persons 60 years of age and above.^3^ According to the National Census of Nepal, the elderly population, was 2.2 million in 2011^4^ compared to 1.5 million in 2001.^5^ In Nepal, the majority (85%) of elderly people reside in rural areas.^6^

Poor nutrition can lead to reduced immunity, increased vulnerability to disease, impairment in physical and mental state and decreased productivity.^7^ There is an association between malnutrition and high rates of morbidity and mortality as well as increased health care expenditures.^8-10^ Elderly population is more likely to be malnourished due to aging and associated problems.^11,12^

There is insufficient information related to the nutritional status of the elderly in Nepal.^14,15^ This study aims to estimate the prevalence of malnutrition among elderly people living in rural areas of the Kavrepalanchok district.

## METHODS

A descriptive cross-sectional study was conducted in a rural population of Kavrepalanchok district to determine the prevalence of malnutrition among the participants. Ethical approval was taken from the Institutional Review Committee (IRC) of Kathmandu University School of Medical Sciences (Ref No.: 68/19). Informed consent was read and explained to the participants and verbal consent was obtained. The study was conducted from August to October 2019.

The study population comprised elderly people aged 60 years and above and holding permanent resident status at the time of the study, defined as at least one year of residence. Individuals unable to respond due to serious physical or mental illness were excluded from the study.

Sample size was calculated as,


$$\begin{array}{ll} \text{n} & ={\text{Z}}^{\text{2}}\times \text{p}\times \text{q}/{\text{e}}^{\text{2}}\\ & ={\left(1.96\right)}^{2}\times 0.24\times 0.76/{\left(0.05\right)}^{\text{2}}\\ & =280.16 \end{array}$$

Where,

n = sample size
Z = 1.96 at 95% Confidence Interval (CI)
p = prevalence of elderly malnutrition i.e. 24%^16^
q = 1-p
e = margin of error, 5%

Considering a 15% non-response rate, the sample size estimated for the study was 322. The convenience sampling method was used to select the household. Only one eligible participant was selected from each household. If there were more than one eligible participant in a household, we selected one participant using a simple random sampling technique following the lottery method.

The nutritional status was assessed using the translated and well-validated Nepali version of the Mini Nutritional Assessment tool specifically designed for elderly people.^16^ Tool comprises eighteen questions and has four sections: anthropometric measurement, global health and social assessment, dietary assessment, and subjective assessment of health and nutrition. According to the score obtained using the MNA tool, nutritional status was categorized as score ≥ 24 are having normal nutrition, 17 - 23.5 “at risk” of malnutrition, <17 malnourished.17A pre-designed structured interview schedule was used for additional data collection regarding socio-demographic and lifestyle variables including age, sex, ethnicity, religion, educational status, marital status, smoking status, alcohol consumption and type of family.

In four anthropometric assessments, height was measured with a portable standard stature scale, without footwear. The participant stood on a flat surface facing the interviewer with their feet together and heels against the backboard with knees straight. Height was recorded in centimeters. Weight was measured with a portable digital weighing scale. The instrument was placed on a firm, flat surface. Weight was measured with minimum clothes and no footwear. Weight was recorded in kilograms. Body Mass Index (BMI) was calculated using formula weight in kilograms divided by the square of the height in meters. Mid Upper Arm Circumference (MUAC), and calf circumference were measured to the nearest 0.1 cm using a non-stretchable measuring tape.

Participants were categorized into three ethnic groups, namely: Upper Caste, Janjatis and Dalit. These three ethnic groups generally represented higher, medium, and lower social status, with the Dalit representing the most marginalized of all groups.^18,19^

Participants were categorized as illiterate, informal, and literate according to their educational status. Informal education referred to having some literacy skills but no formal education.^16^ According to smoking habits, participants were categorized as never smoker, current smoker (smoking for at least a year), and former smoker (quit over one year ago). Alcohol consumption was defined as: never, infrequent (consumed once in a month), frequent (who consumed once or more in a week).^20^

Data analysis was performed using the Statistical Program for Social Sciences (SPSS) version 23. Sample characteristics were described using mean and standard deviation for continuous variable and percentage for a categorical variable.

## RESULTS

A total of 320 individuals participated in the study (males: 193 (60.3%) and females: 127 (39.7%)). Socio-demographic characteristics of the study population are provided (Table 1).

**Table 1:** Characteristics of the elderly participants (n = 320).

Characteristics		Participants mean±SD
Age		68.23±7.38
BMI		22.54±3.25
		n (%)
Age category	60 to 69	193 (60.3)
	70 to 79	98 (30.6)
	80 and above	29 (9.1)
Gender	Male	193 (60.3)
	Female	127 (39.7)
Ethnicity	Upper caste	213 (66.6)
	Janajati	94 (29.3)
	Dalit	13 (4.1)
Religion	Hindu	276 (86.3)
	Buddhist	43 (13.4)
	Christian	1 (0.3)
Education	Literate	42 (13.1)
	Informal	62 (19.4)
	Illiterate	216 (67.5)
Marital status	Married	248 (77.5)
	Separated	2 (0.6)
	Widowed	70 (21.9)
Type of family	Nuclear	111 (34.7)
	Joint	141 (44.1)
	Extended	68 (21.2)
Smoker	Never	149 (46.6)
	Current	97 (30.3)
	Former	74 (23.1)
Alcohol consumption	Never	230 (71.9)
	Infrequent	63 (19.7)
	Frequent	27 (8.4)

The ages of the participants ranged from 60 to 98 years, with a mean age of 68.23±7.38 years (Mean±SD). A majority of 193 (60.3%) of the elderly was in the age group of 60-69. A majority of the participants, 213 (66.6%) belonged to the upper caste category, were Hindu 276 (86.3%) by religion and were illiterate 216 (67.5%). Most of the study subjects, 248 (77.5%) were married. A total of 97 (30.3%) participants were current smokers and 90 (28.1%) reported to have ever consumed alcohol. The mean BMI of the study participants was 22.54±3.25 (Mean±SD). The prevalence of normal nutrition, risk of malnutrition and malnutrition was 38.7%, 49.7% and 11.6%, respectively.

**Figure 1.**
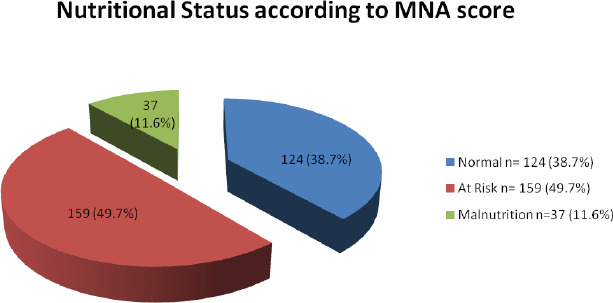
Nutritional status of the respondents according to MNA score.

The nutritional status of the respondents as per the MNA scores. Total 124 (38.7%) had normal nutritional status, 159 (49.7%) were at risk of malnutrition, while 37 (11.6%) of the respondents were malnourished with MNA scores less than 17 points (Figure 1).

**Table 2:** Nutritional status as per the socio-demographic characteristics of the study participants.

	Nutritional Status		
	Normal (24-30)	At Risk (17-23.5)	Malnourished (<17)
	n= 124, 38.7%	n= 159, 49.7%	n= 37, 11.6%
BMIa	23.43±3.18	22.29±3.23	20.66 ± 2.68
Agea	66.13±6.32	68.69±7.29	73.30±8.46
Age	n (%)	n (%)	n (%)
60 - 69 years	91 (47.1)	92 (47.7)	10 (5.2)
70 - 79 years	28 (28.6)	52 (53.1)	18 (18.3)
≥ 80 years	5 (17.3)	15 (51.7)	9 (31.0)
Gender			
Male	94 (48.7)	81 (42.0)	18 (9.3)
Female	30 (23.6)	78 (61.4)	19 (15.0)
Ethnicity			
Upper caste	86 (40.4)	104 (48.8)	23 (10.8)
Janajati	33 (35.1)	48 (51.1)	13 (13.8)
Dalit	5 (38.5)	7 (53.8)	1 (7.7)
Religion			
Hindu	113 (40.9)	133 (48.2)	30 (10.9)
Buddhist	11 (25.6)	25 (58.1)	7 (16.3)
Christian	0 (0.0)	1 (100.0)	0 (0.0)
Education			
Literate	20 (47.6)	17 (40.5)	5 (11.9)
Informal	29 (46.8)	28 (45.2)	5 (8.0)
Illiterate	75 (34.7)	114 (52.8)	27 (12.5)
Marital status			
Married	101 (40.7)	124 (50.0)	23 (9.3)
Separated	2 (100.0)	0 (0.0)	0 (0.0)
Widowed	21 (30.0)	35 (50.0)	14 (20.0)
Type of family			
Nuclear	44 (39.6)	54 (48.7)	13 (11.7)
Joint	57 (40.4)	69 (48.9)	15 (10.7)
Extended	23 (33.8)	36 (53.0)	9 (13.2)
Smoker			
Never	63 (42.3)	75 (50.3)	11 (7.4)
Current	37 (38.2)	46 (47.4)	14 (14.4)
Former	24 (32.4)	38 (51.4)	12 (16.2)
Alcohol consumption			
Never	88 (38.2)	114 (49.6)	28 (12.2)
Infrequent	21 (33.3)	37 (58.8)	5 (7.9)
Frequent	15 (55.6)	8 (29.6)	4 (14.8)
Comorbidities			
Yes	36 (31.6)	58 (50.9)	20 (17.5)
No	88 (42.7)	101 (49.0)	17 (8.3)

Table 2 shows average BMI of the study population in the categories (normal nutrition, risk of malnutrition and malnutrition, respectively) is in the decreasing trend. A greater proportion of females, 19 (15%), were found to be malnourished than males 18 (9.3%).

## DISCUSSION

In this study, we assessed the nutritional status of the elderly population aged 60 years and above using Mini Nutritional Assessment (MNA) Questionnaire. The current study in rural Nepal on 320 elderly participants showed that 11.6% of the elderly were malnourished and about 49.7% of the elderly were at risk of malnutrition. A study from Kerala reported 11.6% were malnourished and 46.5% at risk of malnutrition,^21^ findings from Rajasthan were 11.6% (malnutrition) and 46% (at risk of malnutrition),^22^ studies from Tamilnadu reported 14% malnourished and 49% at risk of malnutrition,^23^ Aliabadi M et al. in their study in Iran reported 12% malnourished and 45.3% at risk of malnutrition.^24^ Our findings are comparable to all of the above-mentioned studies that used MNA as a tool for assessing nutritional status. Compared to our findings, Ghimire et al.^16^ and Tamang et al.,^25^ in their study among Nepal's rural population, reported a higher prevalence of malnutrition (24% and 24.8%, respectively). Similarly, studies conducted in Manipur, West Bengal, Karnataka, and Bangladesh also found a higher prevalence of malnutrition (20.8%, 29.4%, 22.6%, and 26%, respectively).^20,26-28^ There are studies conducted in Pakistan,^29^ India^30^ and Hong Kong^31^ which found a much lower prevalence of malnutrition (5.5%, 7.3% and 1.1% respectively) than our study. This discrepancy may be due to differences in the socioeconomic conditions of the study population.

In our study, we found that the prevalence of malnutrition increased with the increase in age. Similar findings have been reported in the previous studies.^20,23,26,29,32-35^ This observation could be because, with the aging, cumulative effect of many factors like physical inactivity, loss of appetite, presence of other diseases, and adverse economic conditions can contribute to malnutrition in elderly people.

Consistent with our finding, several studies from different parts of the world have found a high prevalence of malnutrition in elderly females than males.^16,20,24,26,28,32,36^ In contrast to our finding, Ramya MS et al. in their study reported that the prevalence of malnutrition was more in males.^34^ Higher prevalence of malnutrition in our findings could be because of the cultural practices in Nepal. According to the tradition, women are the ones to eat last after feeding men and children with nutritious food. At times women may be left with little food and which may be less nutritious. However, we think more detailed studies should be carried out to gather more evidence to support this finding.

Our findings that the prevalence of malnutrition was high among participants who reported to have comorbidities are consistent with the results of earlier studies conducted in Nepal,^16^ Bangladesh,^28^ Pakistan^29^ and Iran.^37^

There were certain limitations of the study; however, this being a cross-sectional study establishing a causal relationship was not possible. The study's findings cannot be generalized as it covered only a sample of individuals from one community.

## CONCLUSIONS

Based on our findings, we can conclude that prevalence of malnutrition and at risk of malnutrition in the study, the population is 11.6% and 49.7%, respectively. Our study found that the prevalence of malnutrition was high among the older age group, female gender and participants with co-morbidities. Interventions to improve the nutritional status of the elderly should focus primarily on older people, the female gender and those who have co-morbidities.
